# Lung surfactant reduces *Staphylococcus aureus* cytotoxicity and protects host immune cells from membrane damage

**DOI:** 10.1128/spectrum.01386-24

**Published:** 2025-04-16

**Authors:** Maria Predtechenskaya, Corbin J. Arbizzani, Sofia R. Shomento, Timothy R. Borgogna, Jovanka M. Voyich

**Affiliations:** 1 Department of Microbiology & Cell Biology, Montana State University, Bozeman, Montana, USA; 2 University of Washington, School of Medicine, Seattle, Washington, USA; University of Dundee, Dundee, United Kingdom

**Keywords:** *Staphylococcus aureus*, surfactant, toxin, gene expression, neutrophils

## Abstract

In this study, we identify that lung surfactant significantly reduces the cytotoxicity of *Staphylococcus aureus* (*S. aureus*) membrane-damaging toxins. Data demonstrate that natural surfactants from mice and rats and commercially available surfactant, Infasurf, protect human primary cells (neutrophils and peripheral blood mononuclear cells) from cytolytic activity caused by *S. aureus* supernatants. Supernatants from *S. aureus* grown in surfactant showed a significant reduction in plasma membrane damage against primary human cells as compared to supernatants grown without surfactant. This reduction was not due to a direct bactericidal effect of the surfactants on *S. aureus* growth. Rat and mouse surfactants downregulated the gene expression of *saeR*, the response regulator of the *S. aureus* two-component system SaeR/S that is responsible for the production of virulence factors which are important during lung infection and cause membrane damage in host cells. Rat and lung surfactants also reduced transcript abundance of SaeR/S-regulated genes *lukF*-PV, *hla*, and *hlgA*. Interestingly, the commercially available surfactant Infasurf did not recapitulate the effect of natural surfactants and did not decrease gene transcription of the virulence genes tested. These data suggest that components of natural surfactants protect lungs from *S. aureus* by suppressing *S. aureus* virulence factors and have implications for the role of surfactants in host defense against *S. aureus*.

**IMPORTANCE:**

This study explored the influence of lung surfactants on membrane-damaging *Staphylococcus aureus* (*S. aureus*) toxins. We demonstrate that natural and commercially available lung surfactants minimize the cytolytic capacity of *S. aureus* supernatants against primary human cells. Data indicate that cytolytic reduction by mouse and rat surfactants was partially due to surfactants reducing transcript abundance of virulence factors. This work identifies a novel role for surfactants and suggests their importance in modulating the severity of *S. aureus* lung infections.

## INTRODUCTION

Pulmonary surfactant is a lipid-rich complex within the alveoli that prevents atelectasis during respiration by maintaining surface tension at the air-liquid interface (1, 2). Lung surfactant is predominantly produced by alveolar type II epithelial cells and is composed of 90% lipids, of which dipalmitoylphosphatidylcholine (DPPC) is the most abundant (3). The remaining 10% are proteins (1–4). There are four surfactant proteins, SP-A, SP-B, SP-C, and SP-D, which have been demonstrated to be essential for the structure and functionality of surfactants (2, 5, 6). SP-A and SP-D lung surfactant proteins are also opsonins that assist phagocytosis of bacterial pathogens by alveolar macrophages and neutrophils (7). SP-A and SP-D have also been shown to increase membrane permeability in Gram-negative bacteria leading to lysis (8).

Lung surfactant components have additional immunomodulatory properties, including anti-angiogenesis activity (9) and inhibition of the generation of reactive oxygen intermediates in neutrophils and monocytes (10). Vesicles containing lung surfactant components, like DPPC, have been shown to decrease macrophage inflammation by inducing expression of innate immune receptors, including Fc receptors, CD11b, scavenger and mannose receptors, and complement receptor CR1 or interfering with toll-like receptor-mediated inflammatory responses (3). It follows that aberrant levels of lung surfactant have been associated with many respiratory problems, among which are pulmonary fibrosis, cystic fibrosis, and chronic obstructive pulmonary disease (COPD) (3), as well as during infection, for example, influenza A virus (11). During lower respiratory infections with influenza A virus, alveolar type II cells are preferentially targeted (12). Infection of these cells leads to measurable disruptions in surfactant production (13).

In a previous study, we identified that antecedent influenza A virus infection in mice increased virulence gene expression in *S. aureus* compared to virulence gene expression during *S. aureus* lung infection only (14). The same study demonstrated that secondary *S. aureus* pneumonia following influenza A viral infection was SaeR/S-dependent (14). These observations combined with previous observations of the natural properties of surfactant provided the basis for the current study to investigate whether surfactant may directly impact virulence gene expression and cytotoxicity of *S. aureus*. Our results demonstrate that the presence of natural as well as commercially available lung surfactants prevent membrane damage in neutrophils and peripheral blood mononuclear cells by *S. aureus* toxins. Murine and rat lung surfactants repressed transcription of *S. aureus* secreted toxins, whereas commercially available surfactants did not decrease transcripts of virulence genes tested. This work adds to our knowledge of the role of surfactants in healthy lungs and implies that lung surfactant contributes to reducing the pathogenicity of bacterial infections.

## MATERIALS AND METHODS

### Bacterial strains and culture conditions

*Staphylococcus aureus* (*S. aureus*) PFGE-type USA300 strain LAC (15) was used in all experiments. Unless noted otherwise, overnight and sub-cultured bacteria (1:100 dilution of overnight) were grown in 20 mL of tryptic soy broth (TSB; EMD Millipore) supplemented with 0.5% glucose with shaking (250 rpm) at 37°C. Optical density at 600 nm (OD_600_) was measured using a NanoDrop 2000c Spectrophotometer (ThermoFisher Scientific), and colony-forming units (CFUs) were determined by plating diluted samples on tryptic soy agar (TSA; EMD Millipore) and enumerated the following day.

### Lung surfactant extraction and growth assays

Pulmonary surfactant isolation was performed using an adaptation of the method described in Inselman et al. (16). Briefly, murine (C57BL/6) or rat (F344BN) lung tissue (0.2 g) was homogenized in 3 mL of ice-cold Dulbecco’s phosphate-buffered saline (DPBS) using a tissue grinder. Homogenate slurry was passed through a 70 µm cell strainer followed by centrifugation at 300 × *g* for 10 minutes at 4°C. The supernatant was collected and placed in microcentrifuge tubes for centrifugation at 18,000 × *g* for 30 minutes at 4°C. The resulting supernatant was aspirated and discarded. The remaining surfactant pellet was resuspended in 1 mL of DPBS at 60°C. Research-grade Infasurf was purchased from Onybiotech. For experiments investigating growth in surfactant, 1 mL of surfactant (at varied concentrations) was added to 4 mL of TSB pre-warmed to 37°C and inoculated with 50 µL of overnight culture. For experiments, adding 1 mL of the original surfactant pellet to 4 mL of TSB was considered 100% surfactant. *S. aureus* cultures were incubated with surfactant for 5 hours at 37°C with shaking (250 rpm). Heat inactivation of proteins was performed by incubating surfactants for 30 minutes at 56°C (17).

### Relative quantitative real-time RT-PCR

Transcription of *S. aureus* genes was assessed using TaqMan real-time reverse transcriptase-PCR (RT-PCR) as previously described (18–20). Briefly, sub-cultured strains were harvested at mid-exponential (ME; OD_600_ = 1.5) or early stationary (ES; OD_600_ = 3.0) phase of growth, mechanically disrupted using a FastPrep FP120 cell disrupter (ThermoFisher Scientific), and RNA purified using RNeasy Kit (Qiagen) as described in reference 18. TaqMan real-time RT-PCR was performed using primer and probe sets as published previously (14) and analyzed with the delta-delta Ct method.

### Human PMN or PBMC plasma membrane integrity assays

Heparinized venous blood from healthy donors was collected in accordance with a protocol approved by the Institutional Review Board for Human Subjects at Montana State University. All donors provided written consent to participate in the study. Human polymorphonuclear leukocytes (neutrophils or PMNs) and/or peripheral blood mononuclear cells (PBMCs) were isolated under endotoxin-free conditions (<25.0 pg/mL) and cell viability and purity of preparations were assessed using a FACSCalibur Flow cytometer (BD Biosciences) as described in references 18 and 21. Assays of intoxicating PMNs with extracellular *S. aureus* proteins were performed as previously described (22, 23). Briefly, supernatants from *S. aureus* sub-cultured for 5 h in TSB with glucose were sterile-filtered (0.22 µm, Avantor) and diluted in DPBS. PMNs or PBMCs (1 × 10^6^) were exposed to varied dilutions of *S. aureus* supernatant. Samples were incubated at 37°C for 60 min then stained with propidium iodide (PI; ThermoFisher Scientific) following the manufacturer’s protocol and then analyzed by FACS as in reference 23.

## RESULTS

### Surfactants from mouse and rat lungs protect immune cells from *S. aureus* toxin-mediated membrane damage

**Fig 1:**
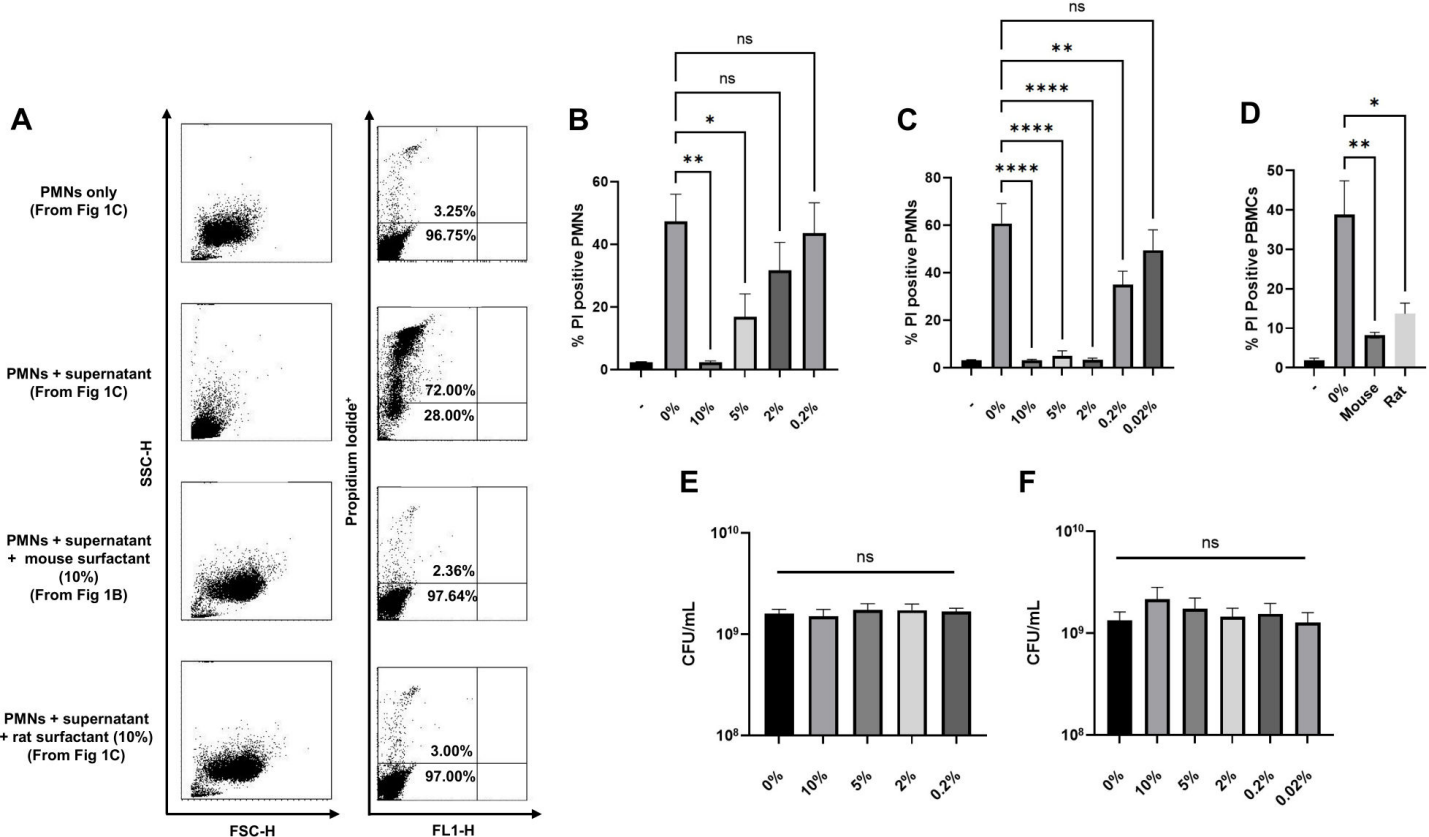
Surfactant from mouse and rat lungs protects human primary cells from *S. aureus*-mediated cytotoxicity. (**A**) Representative flow cytometry plots of data displayed in (B) and (C). *S. aureus* was grown to the early stationary phase in TSB with varied dilutions of (B) mouse or (C) rat lung surfactant. *S. aureus* supernatants were harvested, diluted to 1:50 final concentration, and incubated with human PMNs for 1 hour. Plasma membrane damage was assessed using propidium iodide (PI) uptake and analyzed by flow cytometry. (**D**) Human PBMCs exposed to *S. aureus* supernatants diluted to 1:5 final concentration following growth in 10% mouse or 2% rat surfactant (concentration determined in B and C), and plasma membrane damage assessed with PI. *S. aureus* CFUs collected after growth in mouse (**E**) or rat surfactants (**F**). Dash (-) represents cells without exposure to supernatants or surfactants, while 0% represents cells with exposure to only supernatants. Data are from three biological replicates for (B), (**D**), and (E), and six biological replicates for (C) and (F). *P < 0.05, **P < 0.005, and ****P < 0.0001 one-way ANOVA followed by Dunnett’s multiple comparison test. NS = not significant.

To test the hypothesis that surfactant influences *S. aureus* virulence, we investigated the role of surfactant on *S. aureus* toxin cytotoxicity. In these assays, we compared plasma membrane damage in human neutrophils (PMNs) and peripheral blood mononuclear cells (PBMCs) following exposure to *S. aureus* supernatants grown in the presence or absence of varied concentrations of mouse or rat lung surfactant (Fig. 1A through D). PMNs exposed to supernatants grown in 10% mouse surfactant demonstrated a significant reduction in plasma membrane damage and had an average of 2.39% ± 0.39% propidium iodide (PI)-positive cells compared to 47.39% ± 8.63% PI-positive cells from supernatants grown in TSB only (Fig. 1B). *S. aureus* grown in rat surfactant also demonstrated significantly reduced plasma membrane damage, for example, 3.17% ± 0.33% PI positive in 10% rat surfactant compared to PMNs exposed to supernatants from *S. aureus* grown in TSB only at 60.67% ± 8.44% PI-positive (Fig. 1C). However, unlike mouse surfactant, rat surfactant maintained its ability to significantly reduce plasma membrane damage at a concentration as low as 0.2%. Similarly, growth in mouse or rat surfactants significantly reduced PBMC plasma membrane damage from *S. aureus* supernatants. PBMCs exposed to supernatants harvested from *S. aureus* grown in 10% mouse surfactant or 2% rat surfactant were 8.26% ± 0.72% and 13.70% ± 2.68% PI-positive, respectively, compared to control at 38.90% ± 8.50% PI-positive (Fig. 1D). The reduction of membrane damage in cells was not due to decreased bacterial growth in the presence of surfactants, and no significant differences in bacterial growth were seen in cultures grown with or without surfactant (Fig. 1E and F).

### Commercially available surfactant Infasurf protects immune cells from *S. aureus* toxin-mediated membrane damage

**Fig 2:**
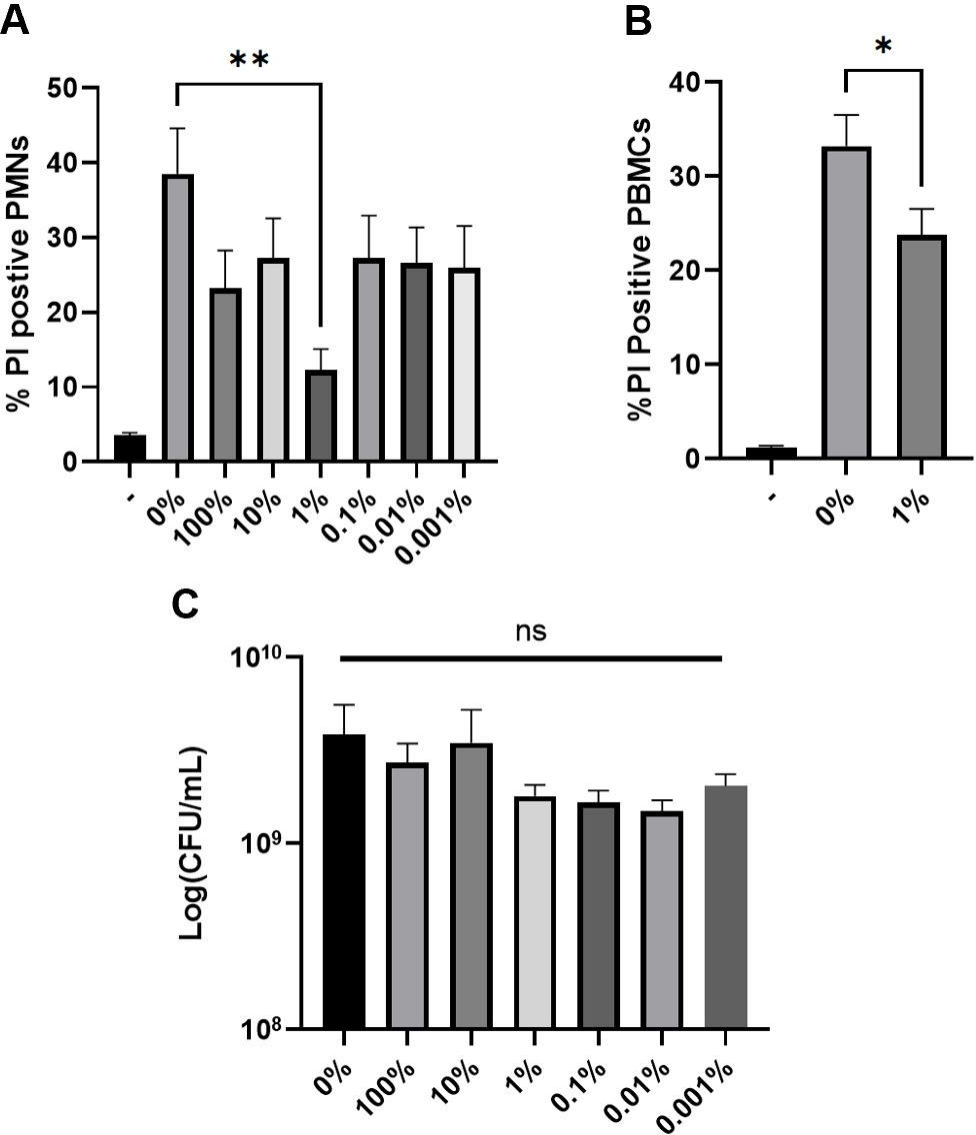
Commercially available surfactant Infasurf protects human primary cells from *S. aureus*-mediated cytotoxicity. (**A**) *S. aureus* was grown to early stationary phase in TSB with varied dilutions of Infasurf and *S. aureus* supernatants were harvested, diluted to 1:50 final dilution, and incubated with human PMNs for 1 hour. Plasma membrane damage was assessed by PI staining and flow cytometry. (**B**) Human peripheral blood mononuclear cells were exposed to *S. aureus* supernatants harvested as in (**A**) following growth in 1% Infasurf (determined in A), and plasma membrane damage was assessed. (**C**) *S. aureus* CFUs collected after growth in Infasurf. Dash (-) represents cells without exposure to supernatants or surfactants while 0% represents cells with exposure to only supernatants. Data are from five biological replicates for (A) and (C), and eight biological replicates for (B). *P < 0.05 and **P < 0.005 one-way ANOVA followed by Dunnett’s multiple comparison test for (A), (B), and (C). NS = not significant.

We next assessed whether this same protective effect could be observed with commercially available surfactants. For these experiments, *S. aureus* was grown in varied concentrations of Infasurf. As above, supernatants were harvested and plasma membrane damage in PMNs and PBMCs was assessed. Only growth in 1% Infasurf provided significantly reduced PI uptake by PMNs. Growth in 1% Infasurf yielded an average of 12.31% ± 2.79% propidium iodide (PI)-positive cells, statistically decreased compared to 38.53% ± 6.05% PI-positive cells from supernatants grown in TSB only (Fig. 2A). This concentration of Infasurf also reduced plasma membrane damage of PBMCs. Without surfactant, PBMCs were 33.21% ± 3.31% PI-positive when exposed to supernatants versus 23.80% ± 2.75% PI-positive with 1% Infasurf (Fig. 2B). As with mouse and rat surfactants, growth in Infasurf did not impact bacterial viability (Fig. 2C). We originally expected that Infasurf would mirror natural surfactants in that higher concentrations would be able to protect immune cells in a dose-dependent manner. Surprisingly, higher Infasurf concentrations no longer protected PMNs from membrane damage from *S. aureus* supernatants (Fig. 2A). Since a previous report of another commercially available surfactant, Surfactant TA observed changes in PMNs consistent with apoptosis (24), we investigated if higher concentrations of Infasurf impacted PMN plasma membrane damage. Incubating PMNs with higher concentrations of Infasurf (without any *S. aureus* supernatants) did not increase PI uptake in PMNs over controls (Fig. S1).

### Mouse and rat lung surfactants modulate *S. aureus* gene expression

**Fig 3:**
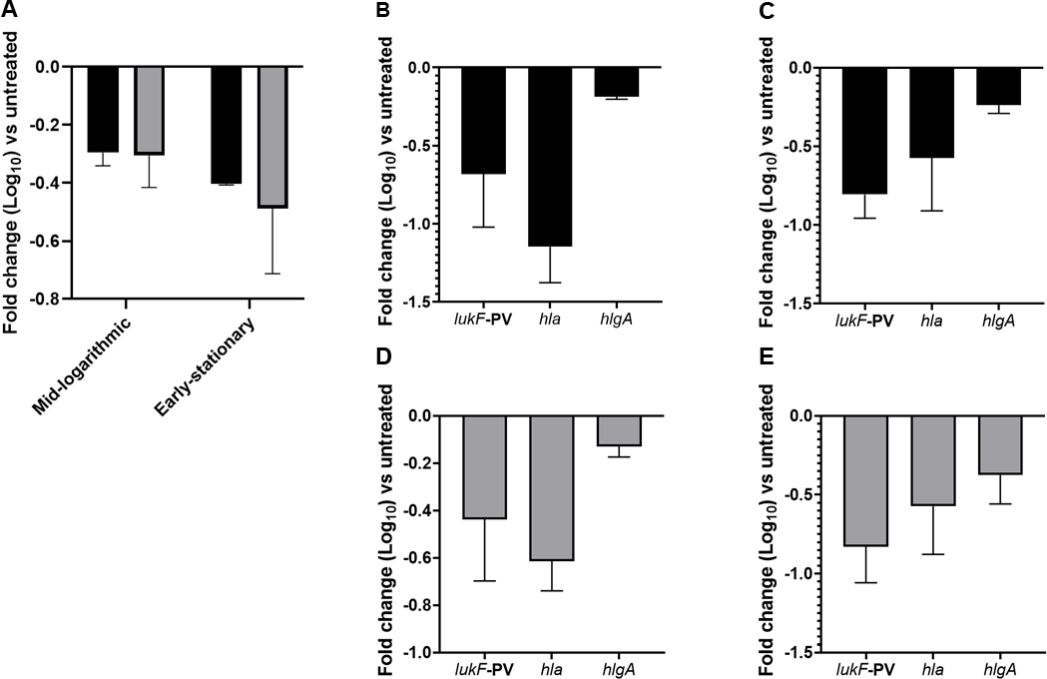
Lung surfactants from mice and rats impact transcription of *S. aureus saeR* and decrease transcription of *S. aureus* virulence genes. (**A**) *S. aureus* was grown in mouse (black) or rat (gray) surfactant to mid-logarithmic or early stationary phase. RNA was harvested and subjected to TaqMan RT-PCR. Gene transcripts were normalized to *gyrB*. Data shown are the mean fold-change of *S. aureus saeR* relative to treatment with *S. aureus* only. For B-E, *S. aureus* was grown in mouse (B and C, black bars) or rat surfactant (D and E, gray bars) to the mid-logarithmic phase (**B and D**) or the early stationary phase (**C and E**) of growth. RNA was harvested as in A. Data shown are the mean fold-change of the indicated gene relative to treatment with *S. aureus* only. Error bars indicate the mean ± SEM of three biological replicates for each surfactant tested.

The SaeR/S system is a two-component system of *S. aureus* responsible for controlling virulence gene expression (23, 25–27). In addition, we and others have previously identified a role for SaeR/S in *S. aureus* lung infections (14, 26). In the current study, *S. aureus* was grown to mid-logarithmic and early stationary phases of growth with or without surfactant. The relative fold decrease in *saeR* compared to control was: −0.29 ± 0.04 for mouse surfactant and −0.30 ± 0.11 for rat surfactant at mid-logarithmic phase (Fig. 3A shown in log scale). The transcript was also reduced at the early stationary phase (−0.40 ± 0.01 for mouse surfactant and −0.48 ± 0.22 for rat surfactant (Fig. 3A).

We next assessed the transcript abundance of *lukF-PV*, *hla*, and *hlgA*, three *S*. *aureus* virulence genes directly regulated by SaeR (21) and demonstrated to be differentially regulated during murine lung infections (14). Compared to control treatment with DPBS, we observed decreases in the abundance of transcripts encoding various toxins when *S. aureus* was grown to either mid-logarithmic or early stationary phase with mouse and rat surfactants (Fig. 3B through E shown in log scale). At mid-logarithmic phase, the relative fold decrease in lukF-PV, hla, and hlgA compared to control for mouse surfactant was: −0.68 ± 0.33,–1.14 ± 0.23,–0.18 ± 0.01, respectively (Fig. 3B). Similarly, the relative fold decrease in *lukF-PV*, *hla*, and *hlgA* compared to control for rat surfactant was: −0.43 ± 0.26, –0.61 ± 0.12, and –0.12 ± 0.04 (Fig. 3D). At early stationary phase, the relative fold decrease in *lukF-PV*, *hla*, and *hlgA* compared to control for mouse surfactant was: −0.80 ± 0.15, –0.57 ± 0.33, –0.23 ± 0.05 (Fig. 3C). For rat surfactant, the relative fold decrease in *lukF-PV*, *hla*, and *hlgA* compared to control was: −0.83 ± 0.22, –0.57 ± 0.30, –0.37 ± 0.18 (Fig. 3E).

### Infasurf modulates gene expression in *S. aureus* but patterns are distinct from natural surfactants

**Fig 4:**
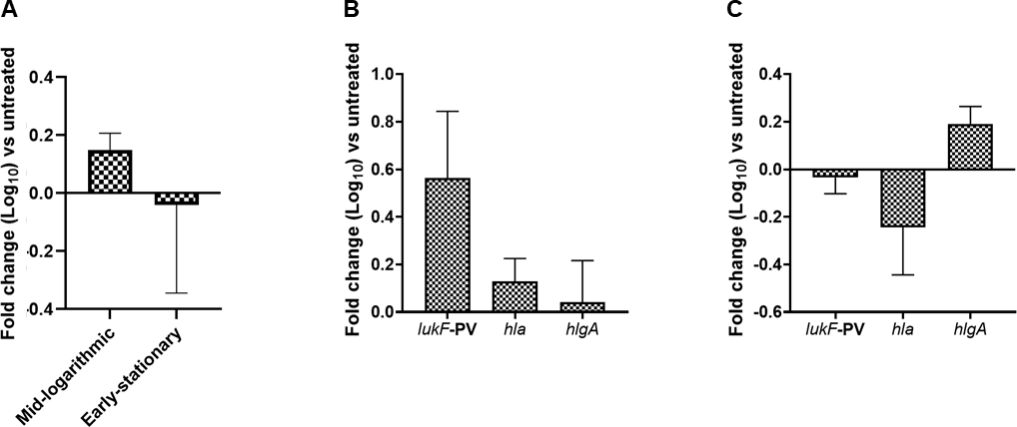
Commercially available surfactant Infasurf impacts transcription of *S. aureus saeR* and *S. aureus* virulence genes. (**A**) *S. aureus* was grown in Infasurf to mid-logarithmic or early stationary phase. RNA was harvested and subjected to TaqMan RT-PCR. Gene transcripts were normalized to *gyrB*. (**A**) Data shown are the mean fold-change of *S. aureus saeR* relative to treatment with *S. aureus* only. *S. aureus* was grown to mid-logarithmic (**B**) or early stationary phase (**C**) of growth in TSB with 1% Infasurf. RNA was harvested as in A. Data shown are the mean fold-change of the indicated gene relative to treatment with *S. aureus* only. Error bars indicate the mean ± SEM of three biological replicates for each surfactant tested.

Next, we investigated whether modulation of gene transcription occurred in *S. aureus* following growth in Infasurf (Fig. 4A through C shown in log scale). Compared to natural surfactants, which had a stable repression of *saeR* in both mid-logarithmic and early stationary phases, commercially available surfactants had a moderate upregulation of *saeR* during the mid-logarithmic phase and a weak downregulation by the early stationary phase. Following growth in 1% Infasurf, the relative fold change of *saeR* compared to control was as follows: +0.14 ± 0.05 in the mid-logarithmic phase and −0.04 ± 0.30 in the early stationary phase (Fig. 4A). In contrast to the results seen with natural surfactant, the presence of Infasurf upregulated *S. aureus* virulence gene transcripts by mid-logarithmic phase. When grown in Infasurf to mid-logarithmic phase, *lukF*-PV, *hla*, and *hlgA* expression were upregulated compared to control by +0.56 ± 0.28, +0.12 ± 0.09, +0.04 ± 0.17, respectively (Fig. 4B). This upregulation of toxin genes during mid-logarithmic phase was consistent with the upregulation of *saeR* observed following growth in Infasurf to mid-logarithmic phase. When grown in Infasurf to early stationary phase, there was a slight reduction in *lukF*-PV and *hla* compared to control: −0.03 ± 0.06 and –0.24 ± 0.20 (Fig. 4C). However, *hlgA* was increased in the presence of surfactant (+0.19 ± 0.07) (Fig. 4C).

## DISCUSSION

This study reports an interesting observation that natural lung surfactants protect host immune cells from damage from *S. aureus*-secreted toxins. We demonstrated that when *S. aureus* is grown in the presence of mouse and rat surfactants, there was a significant decrease in the membrane-damaging ability of secreted *S. aureus* toxins against human PMNs and PBMCs (Fig. 1). To investigate whether the decrease in cytotoxicity in PMNs and PBMCs was regulated at the level of transcription, we assessed gene expression of *saeR*, the response regulator of the two-component gene regulatory system SaeR/S. SaeR/S regulates numerous adhesins, toxins, and immunomodulatory proteins important in neutrophil evasion (28) and is important in mouse models of *S. aureus* lung infections (14, 26). We have previously demonstrated a major role for SaeR/S in the secretion of factors that are cytolytic toward human PMNs (23). In addition, we showed that secondary *S. aureus* pneumonia following influenza A viral infection is SaeR/S-dependent (14). In the current study, we demonstrated that growth of *S. aureus* to mid-logarithmic and early stationary phases in the presence of mouse and rat surfactants decreased *saeR* transcript abundance (Fig. 3). Genes regulated by SaeR/S that encode proteins associated with plasma membrane damage (29) and associated with *S. aureus* lung infections (14, 26) were also downregulated, suggesting that surfactants regulate toxin production at the level of gene transcription. SaeR/S is instrumental in regulating virulence transcripts during the growth phases we tested (19, 20). However, SaeR/S may not be the only two-component system modulated by surfactant. Future studies will investigate other regulatory systems known to regulate toxin production, such as Agr. Agr is well known to impact toxin production in *S. aureus* (30) and understanding its role in response to exposure to surfactant will advance our understanding of how surfactant is modulating *S. aureus* virulence.

Our observations with Infasurf were inconclusive. While we initially expected to find that commercially available surfactant Infasurf would be equally as protective of immune cells as natural surfactants, this was not the case. Protection of cells from membrane damage by cytolytic toxins was only observed when supernatants were harvested from *S. aureus* after growth in 1% Infasurf (Fig. 2). Supernatants harvested following growth in Infasurf in concentrations over 1% increased membrane damage in cells, and protection was rapidly lost at dilutions below 1%. However, this was not due to a direct effect of Infasurf on membrane integrity (Fig. S1).

Results analyzing virulence transcripts following growth in Infasurf were similarly inconclusive and demonstrated no clear pattern of suppression or activation of the genes investigated. Unlike mouse and rat surfactants, Infasurf did not decrease the transcription of *saeR* or associated virulence factors. Similar to our results, a report published by Ishii et al. tested another commercially available surfactant, Surfacten, which is derived from bovine lungs like Infasurf (31). Although there were differences in experimental conditions, including strain used, growth conditions, and assay used for transcript analysis, the study demonstrated that *lukF*-PV, *hla*, and *hlgA* were not significantly influenced by the surfactant at the late logarithmic phase (31). We conclude that Infasurf may be protecting cells through another mechanism or perhaps the timing of when it impacts virulence gene expression is different than that observed with the mouse and rat surfactants. Of note, at the time of this study, we were unable to obtain other commercially available surfactants for research purposes. However, future studies will investigate additional commercially available surfactants to see whether they may behave more similarly to natural surfactants.

One explanation behind the observed variability in the influence of natural and commercially available surfactants on *S. aureus* virulence gene expression and toxin production may be due to differences in surfactant composition, whether this is from different species or varying concentrations. For example, it is known that rat surfactant has a higher phosphatidylcholine concentration than mouse surfactant, both of which are different in composition from bovine surfactant (32). Specific surfactant lipids that have been shown to attenuate inflammation and alter the host response (33) may be at different concentrations in these surfactants. Besides lipid components, there are reports that lung surfactant proteins SP-A and SP-D are involved in pathogen opsonization (3, 5), puncturing microbial membranes (3, 8), suppressing microbial growth, aiding in detoxifying bacterial LPS, and modulating phagocytosis and inflammatory responses by alveolar macrophages (3, 4). SP-D has also been shown to be able to bind to immune cell receptors, modulate complement activation, and enhance bacterial phagocytosis (5). We performed a preliminary screen to identify if proteins contributed to the protective effect we observed in this study. Heat-inactivated mouse and lung surfactants were as protective as untreated surfactants (Fig. S2). Results from our data using heat-inactivated surfactant suggest a lipid component is responsible for our observations. Ishii et al. also suggested that lipid components in surfactants like palmitate can affect virulence expression of *S. aureus* (31). The authors of that study suggest surfactant components may cause membrane stress, triggering *S. aureus* virulence gene expression through the stress response regulator SigB (31).

Others have shown that surfactant components, including SP-A, SP-D (8), and free fatty acids (34), can be bactericidal. However, our findings were not due to surfactant directly impacting *S. aureus* growth, highlighting that another mechanism is influencing the observed decrease in virulence. Our data suggest that modulation of gene expression by natural surfactants reduces *S. aureus* cytotoxicity. Published observations demonstrate that an exposure of *S. aureus* to an overabundance of fatty acids (e.g., oleic, sapienic, myristic, palmitic, myrsitelaidic, lauric acids, and more) shuts down the SaeR/S system (35, 36) support our findings. *S. aureus* has evolved strategies to incorporate and protect itself from host fatty acids through lipases and the incorporation of exogenous fatty acids that alter the *S. aureus* lipid membrane (37–39). The presence of exogenous fatty acids has also been reported to impact SaeR/S activity (40–43). Future studies will investigate the differences in the composition of surfactants to determine the effectors responsible for our observations of reduced virulence gene expression and cytotoxicity following growth or exposure to surfactants.

In summary, this study identified a putative role for surfactants in the protection of the lung from *S. aureus* infection. Our data suggest that the surfactants may provide a first line of defense in healthy lungs against *S. aureus* infection. This would provide a logical explanation for the observation that despite the high number of individuals who are colonized with *S. aureus* in the nares (28, 44), providing frequent opportunity for exposure to the lungs due to natural aspiration, primary *S. aureus* lung infections are not common. We hypothesize that surfactant suppresses *S. aureus* virulence so the pathogen can be cleared with innate immune defenses. It follows that disruption of surfactant abundance or composition may predispose individuals to *S. aureus* lung infection as occurs following influenza A virus infections (14). Future studies will be conducted to test these hypotheses and identify additional mechanisms behind the influence of surfactants on *S. aureus* virulence.

## SUPPLEMENTAL MATERIAL

Fig. S1 spectrum.01386-24-s0001.tiff Commercially available surfactant Infasurf does not cause plasma membrane damage in human PMNs.

Fig. S2 spectrum.01386-24-s0002.tiff Supernatants harvested following growth of *S. aureus* in heat-inactivated surfactant do not increase cytotoxicity to PMNs.

Supplemental material spectrum.01386-24-s0003.docx Supplemental figure legends.

ASM does not own the copyrights to Supplemental Material that may be linked to, or accessed through, an article. The authors have granted ASM a non-exclusive, world-wide license to publish the Supplemental Material files. Please contact the corresponding author directly for reuse.
