# Supplementary figures and images for: Lung surfactant reduces Staphylococcus aureus cytotoxicity and protects host immune cells from membrane damage
Source: Microbiol Spectr. 2025 Apr 16;13(6):e01386-24. doi: 10.1128/spectrum.01386-24 (PMC12131823; doi:10.1128/spectrum.01386-24)

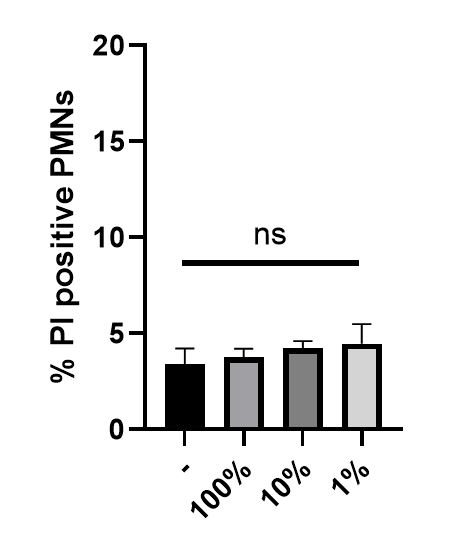

Supplement: Fig. S1 — Commercially available surfactant Infasurf does not cause plasma membrane damage in human PMNs. [file spectrum.01386-24-s0001.tiff]

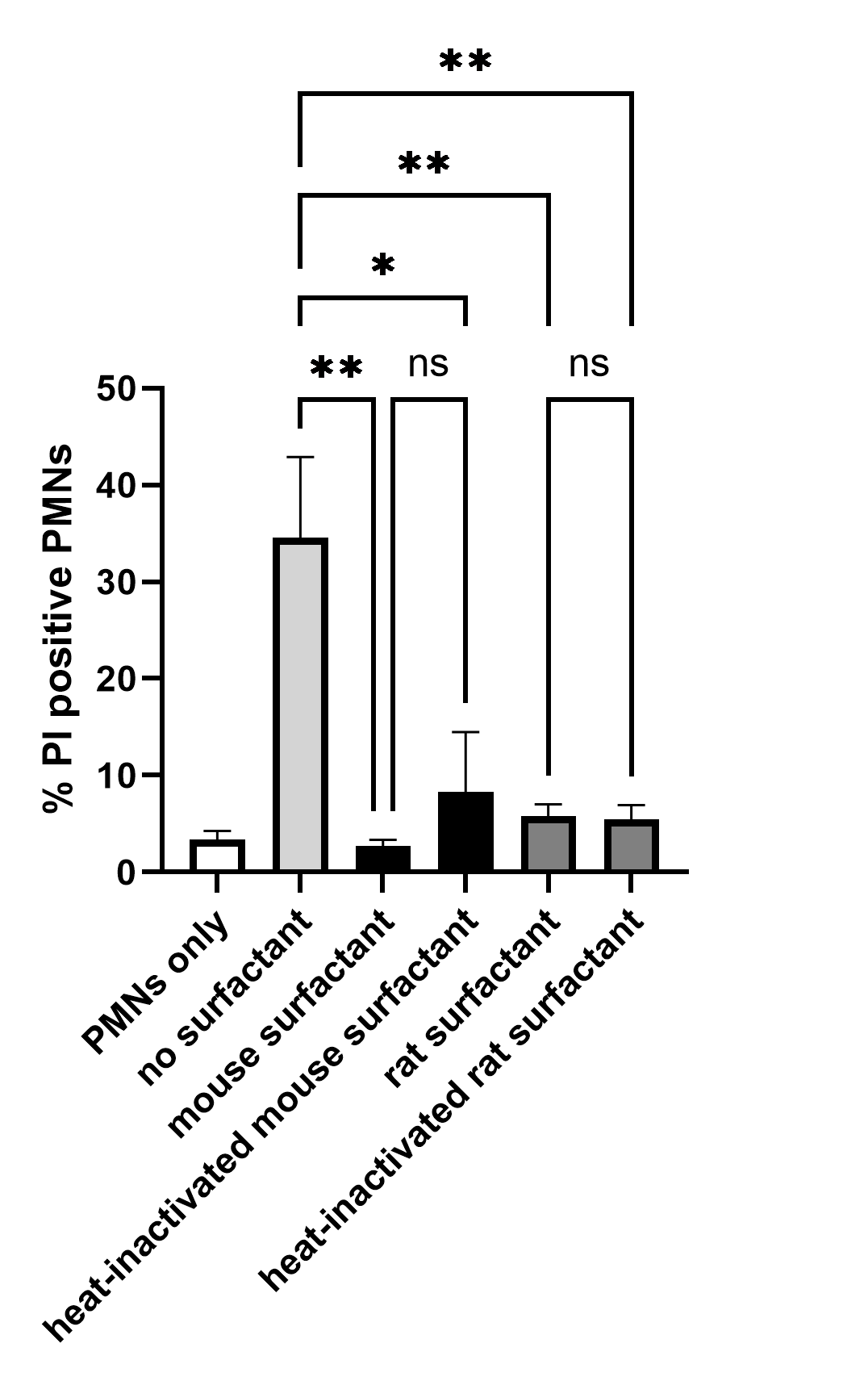

Supplement: Fig. S2 — Supernatants harvested following growth of S. aureus in heat-inactivated surfactant do not increase cytotoxicity to PMNs. [file spectrum.01386-24-s0002.tiff]
