# Supplementary material for: Lung surfactant reduces Staphylococcus aureus cytotoxicity and protects host immune cells from membrane damage
Source: Microbiol Spectr. 2025 Apr 16;13(6):e01386-24. doi: 10.1128/spectrum.01386-24 (PMC12131823; doi:10.1128/spectrum.01386-24)
Supplement: Supplemental material — Supplemental figure legends. [file spectrum.01386-24-s0003.docx]

**Supplemental Figure 1. Commercially available surfactant Infasurf**® **does not cause plasma membrane damage in human PMNs.** TSB with varied dilutions of Infasurf® was incubated for five hours with no bacteria and samples were centrifuged, diluted to 1:50 final dilution, and incubated with human PMNs for one hour. Plasma membrane damage was assessed by PI staining and flow cytometry. Dash (-) represents PMN control without treatment. Data are from 3 biological replicates. NS = not significant by one-way ANOVA followed by Dunnett’s multiple comparison test.

**Supplemental Figure 2: Supernatants Harvested Following Growth of *S. aureus*in Heat Inactivated Surfactant Do Not Increase Cytotoxicity to PMNs.** To heat-inactivate the proteins in surfactants, 10% mouse or 2% rat surfactant, was incubated at 56°C for 30 minutes. *S. aureus* was grown to early stationary phase in TSB with untreated or heat-treated 10% mouse or 2% rat surfactant. *S. aureus* supernatants were harvested, diluted to 1:50 final dilution, and incubated with human PMNs for one hour at 37°C. Plasma membrane damage was assessed by PI staining and flow cytometry. Data are from 3 biological replicates. Error bars indicate mean ± SEM. *P <0.05 one-way ANOVA followed by Tukey’s multiple comparison test. NS = not significant.
